# Comparison of Flavor Compounds in Crab Roe and Paste From Chinese Mitten Crab Raised in Lake, Pond, and Rice-Field Environments

**DOI:** 10.1155/anu/4899168

**Published:** 2025-08-26

**Authors:** Feifei Han, Lulu Zhou, Lu Jin, Yuqing Zhao, Qihan Zhang, Weilin Liu, Jianzhong Han

**Affiliations:** ^1^School of Food Science and Biotechnology, Zhejiang Gongshang University, Hangzhou 310018, China; ^2^Food Safety Key Laboratory of Zhejiang Province, Ministry of Education, Zhejiang Gongshang University, Hangzhou 310018, China

**Keywords:** aquaculture conditions, Chinese mitten crab, crab paste, crab roe, flavor compounds

## Abstract

The Chinese mitten crab (*Eriocheir sinensis*) is an economically important crab species in China, with a unique flavor, excellent nutritional quality and popularity with consumers. However, the current research on Chinese mud crabs predominantly centers on fresh samples and gives limited attention to the flavor characteristics of crabs after steaming. Consequently, this study aims to explore the flavor differences in crab roe and crab paste following steaming across three distinct aquaculture environments: lakes, ponds, and paddy fields. The overall flavor profile was analyzed by electronic nose and electronic tongue. Nonvolatile and volatile substances were determined by HPLC and SPME-GC–MS-O. Subsequently, the taste intensity values (TAVs) and relative odor activity values (ROAV) were calculated. The results demonstrated that the electronic tongue and electronic nose were capable of distinctly discriminating the differences in crab roe and crab juice among the three types of farmed crabs. The levels of umami amino acids and the equivalent umami concentration (EUC) in the roe and paste of pond crabs (PC) were significantly higher compared to those of lake crabs (LC) and rice-field crabs (RC). Significant variations were observed in both the content and types of flavor compounds in the roe and paste of crabs originating from different aquaculture environments. This study provided a basis for improved understanding of the mechanisms of flavor formation in the Chinese mitten crab and provided a basis for regulating its flavor quality.

## 1. Introduction

China boasts the world's largest aquaculture industry, with its annual aquatic product yield comprising more than 60% of the global total. Additionally, the annual yield has been increasing steadily, rising from 7.587 million tons in 2016 to 8.122 million tons in 2023 [1]. The Chinese mitten crab (*Eriocheir sinensis*) commonly known as river crab or hairy crab, is known as one of the most economically important aquaculture species in China [2]. The Chinese mitten crab is widely distributed in the fresh water areas of the Liaohe River, Yellow River, and Yangtze River [3]. Chinese mitten crab is in high demand from consumers and has a high economic value, because of its abundance of essential amino acids, fatty acids, unique taste and pleasant aroma [4–6].

Taste and aroma are the main factors determining the quality and consumer acceptance of Chinese mitten crab, as well as the content and flavor of crab roe and paste. Crab roe refers to the ovaries and hepatopancreas of female crabs, whereas crab paste refers to the male gonadal system and hepatopancreas of male crabs. Nonvolatile taste substances, including free amino acids (FAAs), 5′-nucleotides and betaine make an important contribution to the taste of Chinese mitten crab [4]. The flavor of Chinese mitten crab arises from hundreds of compounds, mainly generated by enzymic- and auto-oxidation of lipids, such as aldehydes, ketones, alkanes, and alcohols [7]. The flavor of Chinese mitten crab is influenced mainly by diet (natural, traditional, or formulated) [8], aquaculture environment (lake, pond, or rice-field) [9] and geographic location [10]. For example, traditional diets produced the greatest abundance of umami amino acids and the highest relative odor activity value (ROAV) in the hepatopancreas, whereas formulated diets had the highest 5′-inosine monophosphate (IMP) concentrations [8]. The content of FAAs in the edible tissues of crabs from different river basins also varies [10]. Wild crabs had a higher free amino acid (FAA) content and more abundant key volatile compounds in the gonads and hepatopancreas than farmed crabs [4]. In recent years, there has been increasing research interest on the influence of aquaculture environment on the sensory quality of Chinese mitten crabs.

The main aquaculture environments for Chinese mitten crab are lake, pond, rice-field and wild-caught. However, the numbers of wild crab are limited and harvesting them is not sustainable [11], so there is increasing interest in farming crabs in lakes, ponds and rice-fields. Pond farming is an intensive, high-density farming method, but produces a high yield [12]. Rice fields have good light, sufficient dissolved oxygen, abundant natural food, less disease and low operating costs. Rice field farming provides a suitable habitat for crabs without reducing rice production and enabling a double harvest of rice and crabs [12, 13]. Lakes have abundant natural plant life, sufficient natural food, low breeding density, and low operating costs. These different aquaculture environments influence the sensory quality of crabs. For example, the flavor of female crab muscle and hepatopancreas from wild crabs was distinct from those of lake (LC) and pond crabs (PC) [14], whereas the fatty acid composition, mineral content and taste of wild crabs were better than those of rice-field crabs (RC) [5]. PC have a higher FAA content in the edible tissue and better flavor than rice field crabs [15]. However, most previous crab sensory quality studies compared only one or two types of aquaculture environment; few studied more than two. In addition, consumers have difficulty distinguishing between the gonads and hepatopancreas when eating crab paste and roe, as they are typically consumed together. Many previous studies have focused solely on individual tissues (gonads, or hepatopancreas), rather than the flavor characteristics of crab paste and roe. Therefore, this study aimed to investigate the flavor characteristics of crab roe and crab meat across three aquaculture environments—lakes, ponds, and rice fields—with the objective of providing scientific guidance for enhancing the quality of crab farming and improving product quality.

## 2. Materials and Methods

### 2.1. Sample Collection and Preparation

Chinese mitten crabs were caught from a PC, a LC, and a RC in Zhejiang province, China and transported to the laboratory. Twenty-four crabs (half male and half female) of average weight 150 g ± 20 g (female) and 200 g ± 20 g (male) from each aquaculture environment were used for experimentation.

The preparation of river crab samples was conducted according to the method described by Mei et al. [15], with appropriate modifications made as needed. The crab were put on ice to render them unconscious, then fully cooked by steaming for 20 min. After cooling to room temperature, the steamed crab roe and crab paste were removed with spoons. Each sample was homogenised and stored at −30°C for further analysis.

### 2.2. Electronic Tongue (E-Tongue) Analysis

The electronic tongue analysis was performed as described previously [16, 17]. After chopping and homogenization, the sample (5 g) was mixed with deionized water 10 mL, then centrifuged (10,000 × g, 10 min, 4°C), the supernatant retained, and the extraction repeated twice. Finally, adjust the volume to 50 mL. The combined extracts were analyzed with the E-tongue (the equipment independently developed by Zhejiang Gongshang University), in triplicate.

### 2.3. Electronic Nose (E-Nose) Analysis

The electronic nose (The equipment independently developed by Zhejiang Gongshang University.) analysis was performed as described previously [18]. Place an exact 2 g sample into a 50 mL headspace vial and maintain it at 60°C for 10 min. Subsequently, withdraw and inject a 2000 µL aliquot into the instrument. The data acquisition duration is 120 s, followed by a 600 s equilibration period after each detection. Each sample is measured in triplicate.

### 2.4. FAA Analysis

The FAA content was analyzed by an automatic amino acid analyzer (L-8900, Hitachi, Japan). Cooked sample (5 g) was vortexed with perchloric acid (10 mL, 5%), then centrifuged (6853 × g, 5 min, 4°C), the supernatant was retained and the extraction repeated. The pH was adjusted to 6.5, the volume adjusted to 50 mL with distilled water and the solution was filtered through a 0.45 μm membrane, before FAA analysis.

### 2.5. The 5'-Nucleotide Analysis

The detection method of 5'-nucleotide was modified based on Zhuang et al. [8]. The cooked sample (5 g) was homogenized in perchloric acid (20 mL, 5%), then centrifuged (4°C, 10,000 × g, 10 min), the supernatant retained, and the extraction repeated. The combined supernatants were adjusted to pH 5.75, diluted to 50 mL with 5% perchloric acid and filtered through a 0.22 μm membrane. HPLC analysis was performed with an UltiMate 3000 (Thermo Fisher Scientific, Waltham, MA, USA) fitted with an Inertsil XSelect Hss T3 column (250 × 4.6 mm, 5 μm; Waters, Milford, MA, USA). The injection volume was 10 μL and the column temperature 25°C. The eluents used were (A) K_2_HPO_3_ (0.01 mol/L, pH 5.75), (B) water, and (C) acetonitrile, at a flow rate was 0.40 mL/min.

### 2.6. Taste Activity Value (TAV)

Taste activity values (TAVs) were calculated as TAV = *C*/*T* (Equation (1) [19] where *C* was the measured concentration of the taste substance and *T* was its taste threshold. TAV was the ratio of the content of each flavor substance in a sample to its taste detection threshold. Generally, compounds with TAV > 1 were considered to be significant contributors to taste.

(1) $$\begin{array}{c} \text{TAV}=C/T. \end{array}$$

### 2.7. Equivalent Umami Concentration (EUC)

The EUC (units g monosodium glutamate [MSG]/100 g sample) is the MSG concentration equivalent to the mixture of umami amino acids and nucleotides in a sample [20]. Equivalent umami concentrations (EUCs) were calculated as follows:

(2) $$\begin{array}{c} \text{EUC}=\sum a_{\text{i}}b_{\text{i}}+\;1218\left(\sum a_{\text{i}}b_{\text{i}}\right)\;\left(\sum a_{\text{j}}b_{\text{j}}\right), \end{array}$$

where *a*_i_ is the umami amino acid (aspartic acid [Asp], glutamic acid [Glu]) concentration (g / 100 g); *a*_j_ is umami 5′-nucleotide concentration 5′-IMP, 5′-guanosine monophosphate (GMP), or 5′-adenosine monophosphate (AMP); b_i_ is the relative umami concentration (RUC) for each amino acid to MSG (Glu, 1; Asp, 0.077); b_j_ is the RUC for each umami 5′-nucleotide to 5′-IMP (5′-IMP, 1; 5′-GMP, 2.3; 5′-AMP, 0.18); and 1218 is the synergistic constant.

### 2.8. Volatile Compounds Analysis

#### 2.8.1. Headspace Solid-Phase Microextraction for Sample Preparation

The sample preparation was based on the methods of Zhao et al. [21] and Wang et al. [22], and adjustments were made accordingly. Briefly, crab sample (60.0 g) was blended until uniform, then an aliquot (5.0 g) put into a 20 mL headspace vial, then the same volume of 15% NaCl solution was added and mixed, followed by addition of internal standard solution (40 μL, 2-octanol, 8.19 μg/mL). The vial was equilibrated with shaking at 60°C for 20 min, then a preconditioned SPME fiber (75 μm CAR/PDMS, Supelco, America) was exposed to the vial headspace for an additional 40 min at 60°C. At the end of the extraction, the fiber was desorbed into the injection port of the GC–MS for 5 min at 250°C. Each sample was extracted in triplicate.

#### 2.8.2. Gas Chromatography–Mass Spectrometry-Olfactometry (SPME-GC–MS-O) for Volatile Compound Analysis

Volatile compounds were detected by solid-phase microextraction gas chromatography-mass spectrometry according to the method of Wu and Wang [23], with some adjustments. Gas chromatography coupled with a mass selective detector (GC 7890B/MS 5977A, Agilent, America), equipped with a DB-WAX capillary column (30 m × 0.25 mm × 0.25 μm, Agilent, America), was used for volatile analysis, with helium as carrier gas at a flow rate of 1 mL/min. The injector temperature was 250°C, in splitless mode. The GC oven temperature program was 40°C for 3 min, increased to 100°C at 4°C/min, maintained for 2 min, then increased to 180°C at 6°C/min, then to 250°C at 10°C/min, maintained for 5 min. Electron impact (EI) ionization (electron energy of 70 eV, detection voltage of 1168 V and mass range 40–400 u) was used, the detector temperature was 230°C and the interface temperature was 250°C. The peak area normalization method was used for relative quantitative calculation to obtain the relative percentage content of each compound.

The carrier gas from the chromatographic column was split equally between the mass spectrometer and the olfactory device (ODP4, GERSTEL, Germany). The temperature of the olfactory transmission line and the olfactory input were 220°C and 100°C, respectively. Three trained sensory panelists recorded the aroma characteristics and retention time of each perceived odor. If the aroma of a substance was recorded by at least two panelists, the substance were considered to be an aroma active component of the crab samples.

### 2.9. Key Volatile Flavor Compounds

The component that contributes the most to the overall flavor of the sample had its ROAVstan defined as 100, and the ROAV of other components (A) was computed in accordance with the formula (3) [24].

(3) $$\begin{array}{c} {\text{ROAV}}_{A}\approx 100\times C_{\text{A}}\times \;T_{\text{stan}}/\left(C_{\text{stan}}\times T_{\text{A}}\right), \end{array}$$

where *C*_A_ represents the relative content of each volatile compound; *C*_stan_ indicates the relative content of the compound which contributes the most to the overall flavor of the sample; *T*_A_ is the sensory threshold corresponding to each volatile component; and *T*_stan_ is the sensory threshold corresponding to the compound that has the greatest contribution to the overall flavor of the sample.

### 2.10. Statistical Analysis

The data were statistically analyzed using one-way ANOVA with SPSS version 20.0. Differences between results were considered significant at *p* < 0.05. Results are expressed as the mean ± SEM. Prism 9 was used for graph drawings [10].

## 3. Results

### 3.1. E-Tongue Data Distribution of Chinese Mitten Crab Roe/Paste in Pond, Lake, and Rice-Field Environments

**Figure 1:**
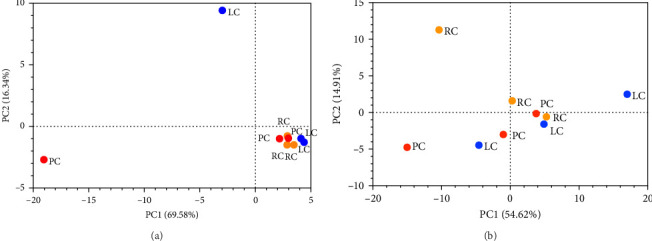
PCA plots of E-tongue taste profiles of Chinese mitten crab from pond, lake and rice-field environments. (A) Crab roe; (B) crab paste. LC, lake crabs; PC, pond crabs; and RC, rice-field crabs.

The taste profile of Chinese mitten crab samples was analyzed using an E-tongue; PCA plots of crab roe/paste data are shown in Figure 1. For crab roe, PC1, and PC2 combined accounted for 85.9% of the differences between samples (Figure 1A). There was no overlap between the roe from PC, LC, and RC, but the separation between LC and PC was small, indicating minor differences between them. For crab paste, PC1, and PC2 combined accounted for 69.5% of the differences between samples (Figure 1B), and PC, LC, and RC were well-separated.

### 3.2. E-Nose Data Distribution of Chinese Mitten Crab Roe/Paste From Pond, Lake, and Rice-Field Environments

**Figure 2:**
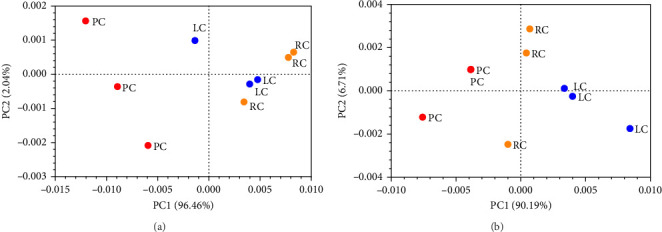
PCA plots of E-nose odor profiles of Chinese mitten crab from pond, lake and rice-field environments. (A) Crab roe and (B) crab paste. LC, lake crabs; PC, pond crabs; and RC, rice-field crabs.

The odor profile of Chinese mitten crab samples was analyzed using an E-nose and PCA (Figure 2). For crab roe, PC1 and PC2 combined accounted for 98.5% of the differences between samples (Figure 2A). There was no overlap between the roe from PC, LC, and RC, indicating that there was a significant difference between them, but the separation between LC and RC was relatively small. For crab roe, PC1 and PC2 combined accounted for 96.9% of the differences between samples (Figure 2B), and PC, LC, and RC were well-separated.

### 3.3. FAA Analysis of Chinese Mitten Crab Roe/Paste From Pond, Lake, and Rice-Field Environments

Seventeen FAAs were detected in crab roe and paste (Table 1). The total FAA contents of crab roe and paste from RC were 34.5 ± 0.08 and 24.8 ± 0.76 mg/g, respectively, higher than those from LC and PC. The sweet amino acid content of the crab roe and paste from RC was higher than those from LC and PC. The bitter amino acid content was higher than that of umami amino acids. The bitter amino acid content of the crab roe and paste from RC was higher than those from LC and PC (*p* < 0.05).

### 3.4. The 5′-Nucleotide Analysis of Chinese Mitten Crab Roe/Crab Paste From Pond, Lake, and Rice-Field Environments

Of the three 5'-nucleotides tested, 5'-AMP was the most abundant in crab roe and paste (Table 2). The AMP and 5'-IMP contents of crab roe from PC were higher than those from LC and RC (*p* < 0.05).

### 3.5. TAV and EUC of Chinese Mitten Crab Roe/Crab Paste From Pond, Lake, and Rice-Field Environments

The TAV and EUC values of umami, sweet and bitter taste compounds in crab roe and paste were calculated to compare the taste profiles of crabs from the three aquaculture environments (Table 3).

A TAV > 1 indicated that the component contributes to the overall taste of the food, and the TAV was positively correlated with the taste contribution of that component. Gly had a TAV < 1 in PC roe (Table 3). GMP had a TAV > 1 in PC, LC and RC roe. Glu, Ala, Arg, Lys and His had TAVs > 1 in PC roe and paste. Gly made the greatest taste contribution in LC and RC roe and paste, whereas AMP made the greatest taste contribution in PC roe. The EUC values of crab roe and paste were different (*p* < 0.05), and the EUC values of PC roe and paste of were higher than those of LC and RC roe and paste (*p* < 0.05).

### 3.6. Volatile Compound Analysis of Chinese Mitten Crab Roe/Crab Paste From Pond, Lake, and Rice-Field Environments

SPME-GC–MS analysis identified 119 volatile compounds in the roe and paste of Chinese mitten crab, of which there were 32 aldehydes, 25 ketones, 15 alcohols, seven aromatic compounds, three furans, nine N-containing compounds, 14 hydrocarbons and 14 other compounds, (Table 4). Of the 119 volatile compounds, 73 and 69 were found in PC roe and paste, 56 and 66 in LC roe and paste, and 66 and 44 RC roe and paste, respectively. Nine compounds [2-ethyl-2-butenal, furfural, 3,5-octadien-2-one, (1-propylnonyl)-benzene, 1,3-diazine, trimethyl-pyrazine, 1-methyl-2-ethylcyclopentene, 1-methoxy-cyclohexene and *n*-caproic acid vinyl ester] were found exclusively in LC roe. Eight compounds (2-methyl-2-butenal, 4-penten-2-ol, 2-butanol, *p*-xylene, cis-2-ethylcyclopentane-carboxaldehyde, 3-methyl-4-propenyl-oxetan-2-one, 1,5-dimethyl-1H-pyrazole and D-limonene) were found exclusively in LC paste. Three compounds [ (E)-2-heptenal, pyridine and dimethyl disulfide] were found exclusively in PC roe and three compounds (1-hydroxy-2-propanone, 6-methyl-5-hepten-2-one and 1-butanol) were found exclusively in PC paste. Two compounds each were found exclusively in RC roe (3-methyl-cyclopentane and 3-methyl-1,2-cyclopentanediol) and RC paste (3-ethyl-2,6,10-trimethylundecane and 2,6,10,14-tetramethyl-pentadecane). The 3-methyl-pentanal and 1,7-dimethyl-7-(4-methyl-3-pentenyl)-tricyclo[2.2.1.0(2,6)] heptane were only found in LC, and dimethyl sulfoxide was only found in RC.

### 3.7. The Key Volatile Flavor Compounds of Chinese Mitten Crab Roe/Crab Paste From Pond, Lake, and Rice-Field Environments Identified by GC–MS

The relative content of the volatile compounds is incapable of reflecting their genuine contribution to the overall aroma characteristics [24]. Therefore, ROAV was utilized to assess the contribution of volatile compounds to the overall aroma profile [25]. The greater the ROAV, the greater the compound's contribution to the overall flavor. Substances with ROAV ≥ 1 are the key flavor compounds of the sample, and substances with 0.1 ≤ ROAV < 1 are the compounds that modify the overall flavor of the sample [24].

The ROAV of volatile flavor components in Chinese mitten crab roe and crab paste from pond, lake and rice-field environments were shown in Table 5. Set the ROAV of 1-Octen-3-one, which contributes the greatest to the overall flavor, at 100. Of the 119 volatile compounds identified, 17 compounds with ROAV > 1 were identified and contributed significantly to the overall flavor of Chinese mitten crab. LC roe and paste had 11 and 9 compounds, PC roe and paste had 14 and 10, and RC roe and paste had 7 and 9, respectively. The *n*-caproic acid vinyl ester was only found in LC roe. Dimethyl Disulfide was only found in PC roe. 4 compounds [pentanal, heptanal, 1-Octen-3-one and 1-Octen-3-ol] were found both in LC, PC and RC roe.

### 3.8. Odor-Active Compound (OAC) Comparison of Chinese Mitten Crab Roe/Paste From Pond, Lake, and Rice-Field Environments

Olfactometric (GC-O) analysis identified 20 OACs (Table 6) out of the 119 volatile compounds identified by the SPME-GC–MS analysis (Table 4). Of the 20 OACs, 14 and 11 compounds were detected in LC roe and paste, 14 and 12 compounds were detected in PC roe and paste, and 15 and 9 compounds were detected in RC roe and paste, respectively. The 2-ethyl-furan, pentanal, hexanal, (Z)-4-heptenal, 1-octen-3-one, 1-octen-3-ol and 2-butanone were present in LC, PC and RC roe and paste. The strongest ROAC was 1-octen-3-one, which has a “metallic, mushroom” aroma. In contrast, ethanethiol, which had a “rotten cabbage” odor, was detected only in PC roe and propanal with a “sharp, musty” odor was detected only in RC roe.

## 4. Discussion

Flavor substances in aquatic products include nitrogen-containing organic compounds, non-nitrogen-containing organic compounds and inorganic ions [26]. FAAs are a subset of the nitrogen-containing organic compounds, mainly contributing umami, sweet and bitter tastes [27, 28]. The umami amino acid content of PC roe and paste was significantly higher than those of LC and RC, but the sweet amino acid content of PC roe and paste was significantly lower than those of LC and RC. RC roe and paste had the highest content of sweet amino acids, which appear to contribute strongly to their characteristic taste. The Gly content of PC roe and paste is lower than LC and RC roe and paste, but the EUC of PC roe and paste was significantly higher than those of LC and RC roe and paste. The sweetness of Gly can mask undesirable odors and enhance umami perception [29], so it appears that umami perception is related to the Gly content in Chinese mitten crab roe and paste.

AMP, IMP, and GMP are 5'-nucleotides with an umami taste [30]. AMP not only masks the bitterness, but also enhances the sweetness and saltiness of aquatic products, and combines with IMP to increase their umami intensity [8]. AMP was the main 5′-nucleotide in the edible tissues of Chinese mitten crab, followed by IMP and GMP, in agreement with previous reports [31, 32]. Although the IMP concentration was relatively low, even a relatively low AMP concentration can contribute a perceptible umami taste and increase sweetness [15]. AMP and IMP were abundant in PC roe and paste, and appear to contribute to their relatively strong umami taste.

The contribution of a flavor substance depends on its TAV in the food matrix [8]. The contents of Glu, Ala, Lys, His and Arg were relatively high in PC, and their TAVs were all > 1, so they all contributed significantly to the taste of PC roe and paste. The TAV of Gly in RC roe and paste was significantly higher than that in LC and PC, and also higher than that of other taste substances, so Gly appears to be the major taste contributor to RC roe and paste. The Glu content of roe and paste from all three aquaculture environments was relatively low, but MSG is an important umami amino acid that works synergistically with IMP to enhance the umami taste, even when the Glu content is below the threshold [10]. The EUC of a food reflects the synergistic effect of MSG and 5′-nucleotides [10]. The TAV of Glu in the roe and paste from the three aquaculture environments was > 1 and the EUC of PC was significantly higher than those from LC and RC, indicating that Glu contributed strongly to the overall umami taste of Chinese mitten crab.

Volatile flavor compounds are low molecular weight compounds that are perceptible by the human odor sensory system. Typically, they are generated by transformation of precursor compounds through processes, such as heating or enzymic catalysis. The precursors of volatile flavors are mainly water-soluble components and lipids, which are converted into aldehydes, ketones, alcohols and other compounds by lipid oxidation, the Maillard reaction and Stecker degradation reaction during cooking [33]. In particular, unsaturated fatty acids, such as oleic acid and linolenic acid, are oxidized during cooking, generating volatile substances, such as ketones and aldehydes. The total volatile compound content in the LC roe and PC paste were significantly higher than those of the other samples. The highest content of “grassy” hexanal was in LC roe, which was significantly higher than those of PC and RC. Aroma formation during cooking of Chinese mitten crab includes the thermal degradation of nonvolatile precursors, such as phospholipids and fatty acids) and the secondary degradation of hydroperoxides [23]. The gonads and hepatopancreas of female crabs after steaming have significantly decreased contents of FAAs and phosphatidyl ethanolamine (PE), with FFA and PE being the main components. PE (18:2/20:4), PE (16:0/20:5), PE (16:0/20:4), and PE (20:5/18:2) are considered to be the main precursors of the aroma of Chinese mitten crab and may be related to the generation of hexanal. Therefore, the lake aquaculture environment appears to favor the accumulation of phospholipids and fatty acids in the crab roe. The free radicals generated by lipid oxidation promote the degradation of amino acid derivatives, producing benzaldehyde. The benzaldehyde content of PC roe was higher than those of LC and RC roe, suggesting greater degradation of amino acid derivatives in the pond-raised crabs.

The content of a volatile substance does not fully reflect its contribution to the overall odor [24], so it is necessary to calculate the ROAV, which relates the content to the sensory detection threshold. The 1-octene-3-one had the highest ROAV of all the volatiles identified in crab roe and paste. The content of 1-octene-3-one was higher in RC roe than in PC and LC roe, and higher in PC paste than in RC and LC paste. Ketones, such as 1-octene-3-one are the products of lipid oxidation, formed in meat products during cooking [14]. 1-octene-3-one was detected in mangrove crab [34] and prawn [35]. Dimethyl Disulfide were detected only in PC roe, and the ROVA of dimethyl disulfide was up to 15.36. Sulfur-containing amino acids can participate in the Maillard reaction to produce thioethers, mercaptans and other sulfur-containing compounds, which have high volatility and appear to contribute strongly to the characteristic flavor of PC roe [36]. 2-emyl-Furan and vinyl n-caproate were detected only in LC roe. Heptanal contribute to the “fresh” aroma of aquatic products, contributing “fruity” aromas, which overlap with many other substances [37]. The 2-Emyl-Furan is a typical oil oxidation product with a “soybean” aroma, which contributes to the overall flavor of Chinese mitten crab; however, this compound can have a negative effect on the flavor of shrimp and crab [38]. Esters are the products of reactions between carboxylic acids and alcohols, generated from fermentation or lipid metabolism, and they generally contribute a “sweet fruity” aroma [39]. Esters appear to contribute strongly to the unique flavor of LC paste and roe. The differences in ROAV among Chinese mitten crab roe and paste from the three different aquaculture environments suggested corresponding differences in their flavor profiles. The differences in odor profile appeared to be the primary contributing factor to the variations in E-nose profiles between them. The data obtained from the electronic tongue and electronic nose axes indicated that the variation in taste across different habitats was more pronounced than the alterations in aroma [40].

The GC–O analysis indicated that 2,6-dimethylpyridine (milk/corn/rice flavor), hexanal (vanilla flavor), (z)-4-heptenal (chemical/bitter flavor), 1-octanol (leafy flavor) and 2-butanone (fatty flavor) in crab roe and paste were detectable by olfaction, even at low concentrations, and contributed to the overall flavor.

## 5. Conclusion

In this study, Chinese mitten crab roe and paste, raised in lake, pond or rice-field aquaculture environments, were compared from the flavor perspective, for the first time. E-tongue and E-nose analysis indicated that Chinese mitten crab from the different aquaculture environments had distinguishable flavor profiles. In terms of nonvolatile taste-active compounds, the FAA and 5'-nucleotide compositions indicated that the TAVs of Glu, Ala, Arg, Lys, and His were > 1 only in the roe and paste from pond-raised crabs, and the EUC of PC roe and paste was greater than those of lake- and rice-field-raised crabs. Therefore, the crab roe and crab paste of pond-raised crabs exhibit a higher degree of freshness, whereas the rice-field-raised crabs demonstrate a higher level of sweetness.

In terms of volatile compounds, 69 and 119 volatile compounds were identified in crab roe and paste, respectively. The total content of volatile substances in crab paste was highest in pond-raised crabs, whereas that in crab roe was highest in lake-raised crabs. However, in all three aquaculture environments, the odor profile of the river crabs was predominantly characterized by the scents of mushrooms and cooking flavor, specifically 1-octen-3. In addition, the primary contributors to the aroma of Chinese mitten crab varied between the different aquaculture environments. Overall, aquaculture environment significantly influenced the flavor of Chinese mitten crab. However, the specific factors influencing their flavor remain unclear. Therefore, investigating the mechanisms influencing taste and flavor profiles from the perspective of amino acid and fatty acid metabolism should be a focus of future research.

## Figures and Tables

**Table 1:** Free amino acid (FAA) content in crab roe/paste from pond, lake, and rice-field environments (mg/g).

Free amino acid composition	Female roe			Male paste		
	LC	PC	RC	LC	PC	RC
Asp	0.01 ± 0.00^b^	0.04 ± 0.00^a^	0.01 ± 0.00^b^	0.01 ± 0.00^b^	0.04 ± 0.00^a^	0.01 ± 0.00^b^
Glu	0.18 ± 0.01^b^	0.42 ± 0.01^a^	0.19 ± 0.00^b^	0.16 ± 0.01^b^	0.41 ± 0.00^a^	0.16 ± 0.00^b^
Umami amino acid	0.18 ± 0.01^b^	0.46 ± 0.01^a^	0.19 ± 0.00^b^	0.16 ± 0.01^b^	0.44 ± 0.01^a^	0.17 ± 0.00^b^

Thr	0.38 ± 0.00^b^	0.42 ± 0.01^a^	0.43 ± 0.00^a^	0.37 ± 0.00^b^	0.41 ± 0.01^a^	0.38 ± 0.00^b^
Ser	0.02 ± 0.00^b^	0.41 ± 0.00^a^	0.02 ± 0.00^b^	0.02 ± 0.00^b^	0.42 ± 0.01^a^	0.02 ± 0.00^b^
Gly	18.92 ± 1.36^b^	1.17 ± 0.22^c^	28.01 ± 0.04^a^	18.14 ± 0.08^b^	1.33 ± 0.01^c^	19.46 ± 0.63^b^
Ala	0.02 ± 0.00^b^	4.05 ± 0.08^a^	0.02 ± 0.00^b^	0.01 ± 0.00	3.81 ± 0.03	0.02 ± 0.00
Arg	0.20 ± 0.01^b^	3.91 ± 0.03^a^	0.18 ± 0.00^b^	0.18 ± 0.01^b^	3.82 ± 0.03^a^	0.16 ± 0.00^b^
Pro	0.74 ± 0.02^c^	1.54 ± 0.02^a^	0.72 ± 0.00^c^	0.69 ± 0.01^c^	1.41 ± 0.01^b^	0.72 ± 0.01^c^
Sweet amino acid	20.28 ± 1.36^b^	11.50 ± 0.30^c^	29.38 ± 0.05^a^	19.40 ± 0.08^b^	11.20 ± 0.06^c^	20.75 ± 0.63^b^

Cys	0.04 ± 0.00^a^	0.02 ± 0.00^b^	0.04 ± 0.00^a^	0.04 ± 0.00^a^	0.01 ± 0.00^b^	0.04 ± 0.00^a^
Val	2.06 ± 0.19^b^	0.44 ± 0.00^c^	2.89 ± 0.01^a^	1.79 ± 0.01^b^	0.42 ± 0.01^c^	1.98 ± 0.08^b^
Met	0.15 ± 0.01^b^	0.28 ± 0.01^a^	0.17 ± 0.00^b^	0.15 ± 0.00^b^	0.26 ± 0.00^a^	0.15 ± 0.00^b^
Ile	0.18 ± 0.00^b^	0.13 ± 0.00^c^	0.22 ± 0.01^a^	0.17 ± 0.00^b^	0.13 ± 0.00^c^	0.17 ± 0.00^b^
Leu	0.29 ± 0.02^b^	0.69 ± 0.01^a^	0.37 ± 0.01^b^	0.31 ± 0.00^b^	0.67 ± 0.01^a^	0.33 ± 0.01^b^
Tyr	0.45 ± 0.03^b^	0.33 ± 0.00^c^	0.53 ± 0.01^a^	0.45 ± 0.00^b^	0.31 ± 0.01^c^	0.48 ± 0.01^ab^
Phe	0.50 ± 0.01^b^	0.38 ± 0.00^c^	0.57 ± 0.01^a^	0.49 ± 0.00^b^	0.39 ± 0.02^c^	0.52 ± 0.01^ab^
Lys	0.17 ± 0.02^b^	0.89 ± 0.00^a^	0.17 ± 0.00^b^	0.13 ± 0.00^b^	0.85 ± 0.01^a^	0.14 ± 0.00^b^
His	0.04 ± 0.00^b^	0.21 ± 0.00^a^	0.05 ± 0.00^b^	0.04 ± 0.00^b^	0.21 ± 0.00^a^	0.04 ± 0.00^b^
Bitter amino acid	3.88 ± 0.26^b^	3.38 ± 0.03^cd^	4.99 ± 0.03^a^	3.56 ± 0.00^c^	3.25 ± 0.02^d^	3.85 ± 0.13^b^

*∑* (mg/g)	24.33 ± 1.58^b^	15.34 ± 0.29^c^	34.56 ± 0.08^a^	23.12 ± 0.09^b^	14.90 ± 0.08^c^	24.76 ± 0.76^b^

*Note:* Data are expressed as mean ± SEM (*n* = 3). Values in the same line with different letters are significantly different (*p* < 0.05). Umami amino acids: Asp and Glu; sweet amino acids: Thr, Ser, Gly, Ala, Arg, and Pro; and bitter amino acids: Cys, Val, Met, Ile, Leu, Tyr, Phe, Lys, and His.

Abbreviations: Ala, alanine; Arg, arginine; Asp, aspartic acid; Cys, cysteine; Gly, glycine; His, histidine; Ile, isoleucine; LC, lake crabs; Leu, leucine; Lys, lysine; Met, methionine; PC, pond crabs; Phe, phenylalanine; Pro, proline; RC, rice-field crabs; Ser, serine; Thr, threonine; Tyr, tyrosine; Val, valine.

**Table 2:** Taste nucleotide content of crab roe/paste from pond, lake and rice-field environments (mg/kg).

Nucleotide composition	Female roe			Male paste		
	LC	PC	RC	LC	PC	RC
GMP	226.46 ± 10.01^ab^	239.93 ± 16.19^a^	205.29 ± 2.33^b^	36.43 ± 2.46^c^	36.66 ± 6.82^c^	40.78 ± 0.61^c^
IMP	75.31 ± 13.92^ab^	100.48 ± 9.86^a^	65.15 ± 1.60^b^	5.71 ± 0.96^c^	11.61 ± 1.28^c^	9.09 ± 1.38^c^
AMP	4491.87 ± 90.88^b^	5278.10 ± 87.39^a^	3697.28 ± 90.81^c^	336.47 ± 9.20^d^	375.26 ± 7.08^d^	226.43 ± 11.63^e^

*Note:* Data are presented as the mean ± SEM (*n* = 3). Values in the same line with different letters are significantly different (*p* < 0.05).AMP, 5-adenosine monophosphate; GMP, 5-guanosine monophosphate; IMP, 5-inosine monophosphate.

Abbreviations: LC, lake crabs; PC, pond crabs; RC, rice-field crabs.

**Table 3:** Taste activity value (TAV) and equivalent umami concentration (EUC) of crab roe/crab paste from pond, lake, and rice-field environments.

Flavour substances	Threshold values(mg/g)	Female roe			Male paste		
		LC	PC	RC	LC	PC	RC
Asp	1	0.01 ± 0.00^b^	0.04 ± 0.00^a^	0.01 ± 0.00^b^	0.01 ± 0.00^b^	0.04 ± 0.00^a^	0.01 ± 0.0^b^
Glu	0.3	0.59 ± 0.02^b^	1.41 ± 0.02^a^	0.63 ± 0.00^b^	0.53 ± 0.02^b^	1.35 ± 0.01^a^	0.54 ± 0.01^b^
Thr	2.6	0.15 ± 0.00^b^	0.16 ± 0.00^a^	0.16 ± 0.00^a^	0.14 ± 0.00^b^	0.16 ± 0.00^a^	0.14 ± 0.00^b^
Ser	1.5	0.01 ± 0.00^b^	0.27 ± 0.00^a^	0.01 ± 0.00^b^	0.01 ± 0.00^b^	0.28 ± 0.01^a^	0.01 ± 0.00^b^
Gly	1.3	14.56 ± 1.05^b^	0.90 ± 0.17^c^	21.55 ± 0.03^a^	13.95 ± 0.06^b^	1.02 ± 0.01^c^	14.97 ± 0.48^b^
Ala	0.6	0.03 ± 0.00^b^	6.75 ± 0.14^a^	0.03 ± 0.00^b^	0.02 ± 0.00^b^	6.36 ± 0.05^a^	0.03 ± 0.00^b^
Arg	0.5	0.40 ± 0.03^b^	7.82 ± 0.07^a^	0.37 ± 0.01^b^	0.35 ± 0.01^b^	7.65 ± 0.06^a^	0.32 ± 0.01^b^
Pro	3	0.25 ± 0.01^c^	0.51 ± 0.01^a^	0.24 ± 0.00^c^	0.23 ± 0.00^c^	0.47 ± 0.00^b^	0.24 ± 0.00^c^
Cys							
Val	0.4	5.15 ± 0.49^b^	1.10 ± 0.01d	7.23 ± 0.00^a^	4.47 ± 0.01^c^	1.05 ± 0.02d	4.95 ± 0.19^b^
Met	0.3	0.51 ± 0.02^c^	0.95 ± 0.04^a^	0.56 ± 0.01^c^	0.49 ± 0.01^c^	0.86 ± 0.01^b^	0.52 ± 0.01^c^
Ile	0.9	0.20 ± 0.00^b^	0.15 ± 0.00^c^	0.24 ± 0.01^a^	0.19 ± 0.00^b^	0.15 ± 0.00^c^	0.19 ± 0.00^b^
Leu	1.9	0.15 ± 0.01^c^	0.36 ± 0.00^a^	0.19 ± 0.00^b^	0.16 ± 0.00^c^	0.35 ± 0.01^a^	0.18 ± 0.01^bc^
Tyr							
Phe	0.9	0.55 ± 0.01^b^	0.42 ± 0.00^c^	0.63 ± 0.01^a^	0.55 ± 0.00^b^	0.44 ± 0.03^c^	0.58 ± 0.01^ab^
Lys	0.5	0.33 ± 0.04^c^	1.78 ± 0.01^a^	0.33 ± 0.00^c^	0.26 ± 0.00^c^	1.69 ± 0.03^b^	0.27 ± 0.00^c^
His	0.2	0.22 ± 0.01^bc^	1.05 ± 0.02^a^	0.24 ± 0.00^b^	0.18 ± 0.00^c^	1.03 ± 0.01^a^	0.19 ± 0.00^c^
GMP	0.125	1.81 ± 0.08^ab^	1.92 ± 0.13^a^	1.64 ± 0.02^b^	0.29 ± 0.02^c^	0.29 ± 0.05^c^	0.33 ± 0.00^c^
IMP	0.25	0.30 ± 0.06^ab^	0.40 ± 0.04^a^	0.26 ± 0.01^b^	0.03 ± 0.00^c^	0.05 ± 0.01^c^	0.04 ± 0.01^c^
AMP	0.5	8.98 ± 0.18^b^	10.56 ± 2.97^a^	7.39 ± 0.18^c^	0.67 ± 0.12^d^	0.75 ± 0.11^d^	0.45 ± 0.02^d^
EUC (mg MSG/g)	—	303.15 ± 7.90^ab^	347.55 ± 5.41^a^	259.60 ± 5.05^b^	29.98 ± 4.24^c^	32.78 ± 5.22^c^	28.07 ± 1.69^c^

*Note:* Data are expressed as the mean ± SEM (*n* = 3). Values in the same line with different letters are significantly different (*p* < 0.05). AMP, 5'-adenosine monophosphate; GMP, 5'-guanosine monophosphate; and IMP, 5'-inosine monophosphate.

Abbreviations: Ala, Alanine; Arg, arginine; Asp, aspartic acid; Cys, cysteine; Glu, glutamic acid; Gly, glycine; His, histidine; Ile, isoleucine; LC, lake crabs; Leu, leucine; Lys, lysine; Met, methionine; PC, pond crabs; Phe, phenylalanine; Pro, proline; RC, rice-field crabs; Ser, serine; Thr, threonine; Tyr, tyrosine; Val, valine.

**Table 4:** Volatile compounds identified in roe/paste from pond, lake and rice-field environments (μg/kg).

Compounds	Odor threshold (μg/kg)	Female roe			Male paste		
		LC	PC	RC	LC	PC	RC
Propanal	37	56.63 ± 9.36^a^	29.41 ± 4.17^c^	47.87 ± 9.77^b^	4.98 ± 0.02^d^	7.41 ± 4.06^d^	6.38 ± 0.93^d^
2-Methyl-propanal	N.A	52.15 ± 11.53^b^	143.01 ± 13.34^a^	26.95 ± 4.76^c^	33.41 ± 6.63^c^	15.34 ± 1.60^d^	18.03 ± 3.13^d^
Butanal	1	24.05 ± 2.27^b^	70.10 ± 10.92^a^	17.39 ± 3.11^bc^	7.05 ± 0.47^d^	22.27 ± 0.47^b^	14.22 ± 1.12^c^
2-Methyl-butanal	1	5.76 ± 0.45^c^	17.63 ± 1.29^a^	2.40 ± 0.55^d^	3.09 ± 0.96^cd^	16.34 ± 1.49^a^	10.61 ± 1.65^b^
3-Methyl-butanal	1.6	24.93 ± 2.50^c^	123.16 ± 5.00^a^	15.19 ± 4.75^d^	12.34 ± 0.98^d^	124.00 ± 22.25^a^	40.26 ± 3.14^b^
Pentanal	9	648.68 ± 45.13^c^	924.14 ± 76.71^b^	455.07 ± 82.48^cd^	181.69 ± 33.79^d^	1619.68 ± 65.77^a^	580.16 ± 80.42^c^
3-Methyl-pentanal	N.A	N.D	32.57 ± 3.05^b^	N.D	N.D	42.55 ± 5.18^a^	N.D
Hexanal	4500	3105.84 ± 357.21^a^	1484.60 ± 70.07^c^	2190.97 ± 303.56^b^	119.10 ± 33.30^d^	211.32 ± 41.28^d^	143.63 ± 19.19^d^
2-Methyl-2-butenal	458.9	N.D	N.D	N.D	1.25 ± 0.22^a^	N.D	N.D
*(E)-2-Pentenal*	1500	120.15 ± 3.44^a^	59.29 ± 3.56^b^	111.34 ± 7.75^a^	32.25 ± 7.15^c^	33.55 ± 4.53^c^	17.16 ± 1.81^d^
2-ethyl-2-butenal	N.A	3.11 ± 0.18^a^	N.D	N.D	N.D	N.D	N.D
Heptanal	2.8	220.73 ± 17.24^a^	156.90 ± 5.16^b^	215.82 ± 33.20^a^	17.04 ± 3.14^c^	25.35 ± 7.96^c^	9.93 ± 2.09^d^
3-Methyl-2-butenal	N.A	7.44 ± 1.42^b^	35.25 ± 2.95^a^	4.86 ± 0.36^c^	3.95 ± 0.86^c^	8.96 ± 3.14^b^	4.14 ± 0.36^c^
(*E*)-2-Hexenal	19.2	122.61 ± 4.27^a^	90.88 ± 4.98^b^	96.39 ± 7.45^b^	31.59 ± 4.19^c^	41.53 ± 10.09^c^	19.11 ± 2.34^d^
(*Z*)-4-Heptenal	4.2	15.39 ± 4.57^a^	6.08 ± 0.88^c^	11.25 ± 0.48^b^	2.81 ± 0.53^d^	4.07 ± 1.46^c^	1.36 ± 0.49^d^
Octanal	0.587	55.59 ± 6.51^a^	50.60 ± 3.80^a^	47.25 ± 0.10^b^	N.D	3.03 ± 0.86^c^	3.18 ± 0.31^c^
cis-2-Ethylcyclopentanecarboxaldehyde	N.A	N.D	N.D	N.D	1.78 ± 0.52^a^	N.D	N.D
(*E*)-2-Heptenal	17	N.D	29.55 ± 2.92^a^	N.D	N.D	N.D	N.D
Nonanal	1.1	53.66 ± 5.80^a^	34.46 ± 2.84^b^	44.91 ± 4.39^ab^	5.24 ± 0.70^c^	6.35 ± 1.59^c^	3.71 ± 0.92^d^
(*E,E*)-2,4-Hexadienal	N.A	6.40 ± 0.38^a^	N.D	5.82 ± 0.30^a^	1.64 ± 0.07^b^	N.D	N.D
5-Ethylcyclopent-1-enecarboxaldehyde	N.A	17.77 ± 2.42^a^	N.D	13.55 ± 0.74^b^	N.D	N.D	N.D
(*E*)-2-Octenal	3	43.53 ± 7.77^a^	15.70 ± 2.59^b^	40.26 ± 1.57^a^	6.39 ± 1.85^c^	6.60 ± 0.44^c^	2.38 ± 0.29^d^
Furfural	3000	1.25 ± 0.30^a^	N.D	N.D	N.D	N.D	N.D
(*E,E*)-2,4-Heptadienal	15.4	5.21 ± 1.84^b^	N.D	9.24 ± 1.79^a^	N.D	11.33 ± 1.23^a^	2.23 ± 0.12^c^
Decanal	2.6	3.59 ± 0.38^a^	N.D	2.48 ± 0.11^b^	N.D	N.D	N.D
Benzaldehyde	41.7	109.27 ± 18.34^b^	189.83 ± 8.85^a^	58.43 ± 5.29^c^	33.05 ± 5.25^d^	90.48 ± 11.01^b^	44.34 ± 2.46^cd^
(*E*)-2-Nonenal	0.08	15.22 ± 4.32^a^	N.D	7.25 ± 0.12^b^	N.D	N.D	N.D
(*E,Z*)-2,6-Noa^a^dienal	0.8	5.94 ± 0.55^a^	N.D	4.90 ± 0.53^a^	1.09 ± 0.17^b^	1.06 ± 0.23^b^	N.D
(*E,E*)-2,4-Octadienal	0.15	2.4 ± 0.335^c^	7.10 ± 0.73^a^	2.62 ± 0.07^c^	N.D	4.10 ± 0.79^b^	N.D
Benzeneacetaldehyde	4	0.87 ± 0.22^b^	4.76 ± 0.77^a^	N.D	N.D	1.34 ± 0.09^b^	N.D
4-Ethyl-benzaldehyde	N.A	2.79 ± 0.61^a^	N.D	1.28 ± 0.07^a^	N.D	N.D	N.D
4-Oxohex-2-enal	N.A	1.93 ± 0.19	N.D	1.40 ± 0.04	N.D	1.41 ± 0.14	N.D
Aldehydes (32)							
2-Butanone	440	42.56 ± 8.75^b^	99.53 ± 8.77^a^	8.50 ± 0.75^d^	29.95 ± 5.59^bc^	23.54 ± 7.28^c^	31.11 ± 9.961^bc^
1-Penten-3-one	1	39.56 ± 10.91^a^	24.32 ± 1.02^b^	N.D	15.35 ± 4.36^c^	23.33 ± 6.94^b^	N.D
2,3-Pentanedione	N.A	245.40 ± 23.97^a^	19.03 ± 1.29^d^	97.63± 16.95^b^	72.65 ± 7.44^bc^	43.42 ± 12.16^c^	55.91 ± 15.71^c^
6-Methyl-2-Heptanone	N.A	4.52 ± 1.32^a^	N.D	3.18 ± 0.51^b^	N.D	N.D	N.D
2-Octanone	50.2	23.81 ± 1.18^b^	42.21 ± 1.57^a^	5.70 ± 1.72^c^	25.44 ± 7.24^b^	30.79 ± 3.74^b^	3.70 ± 0.74^c^
2-Hexanone	140	N.D	11.87 ± 2.37^b^	21.56 ± 1.43^a^	N.D	N.D	N.D
1-Hydroxy-2-Propanone	50	N.D	N.D	N.D	N.D	3.25 ± 1.04^a^	N.D
1-Octen-3-one	0.005	17.01 ± 5.52^a^	8.80 ± 2.21^b^	N.D	2.97 ± 0.68^cd^	3.87 ± 0.36^c^	1.96 ± 0.28^d^
2,3-Octanedione	12	N.D	N.D	66.82 ± 4.32^a^	38.65 ± 2.26^b^	N.D	23.40 ± 4.45^c^
6-Methyl-5-Hepten-2-one	300	N.D	N.D	N.D	N.D	1.41 ± 0.43^a^	N.D
2-Nonanone	38.9	3.09 ± 0.11^b^	N.D	6.25 ± 1.92^a^	1.55 ± 0.33^c^	N.D	N.D
3-Methyl-4-propenyl-oxetan-2-one	N.A	N.D	N.D	N.D	1.93 ± 0.53^a^	N.D	N.D
3,5-Octadien-2-one	150	4.19 ± 0.90^a^	N.D	N.D	N.D	N.D	N.D
Acetophenone	10	N.D	N.D	N.D	0.91 ± 0.10^a^	0.81 ± 0.14^a^	N.D
Spiro [4.5]decan-6-one	N.A	N.D	N.D	N.D	N.D	1.99 ± 0.36^a^	1.32 ± 0.00^a^
Ketones (15)							
Methanethiol	2.1	1.72 ± 0.98^b^	12.86 ± 8.76^a^	2.00 ± 0.29^b^	N.D	N.D	N.D
4-Penten-2-ol	N.A	N.D	N.D	N.D	1.09 ± 0.24^a^	N.D	N.D
2-Butanol	N.A	N.D	N.D	N.D	2.79 ± 0.82^a^	N.D	N.D
3-Methyl-cyclopentano	N.A	N.D	N.D	4.98 ± 0.10^a^	N.D	N.D	N.D
1-Butanol	5000	N.D	N.D	N.D	N.D	1.82 ± 0.40^a^	N.D
1-Penten-3-ol	358.1	182.10 ± 26.29^a^	52.21 ± 11.74^c^d	146.37 ± 0.19^b^	88.88 ± 10.53^c^	33.44 ± 6.44^d^	30.32 ± 7.10^d^
1-Pentanol	150.2	138.39 ± 14.02^b^	111.70 ± 2.18^bc^	91.90 ± 6.16^c^	159.86 ± 41.00^b^	394.73 ± 29.56^a^	152.03 ± 31.43^b^
(*E*)-2-Penten-1-ol	N.A	5.12 ± 0.73^a^	N.D	2.66 ± 0.75^b^	2.82 ± 0.22^b^	N.D	N.D
1-Hexanol	2500	16.08 ± 0.27^a^	11.39 ± 0.32^b^	9.40 ± 0.62^bc^	7.36 ± 1.12^c^	6.44 ± 1.23^c^	N.D
1-Octen-3-ol	1.5	92.85 ± 4.92^a^	86.30 ± 12.34^ab^	66.16 ± 2.08^b^	35.97 ± 1.92^c^	27.26 ± 3.97^c^	13.17 ± 1.04d
Linalool	6	N.D	N.D	N.D	1.74 ± 0.56^a^	N.D	N.D
3,4-Dimethylcyclohexanol	N.A	9.13 ± 1.18^a^	3.55 ± 0.04^b^	2.85 ± 0.39^c^	1.60 ± 0.30^d^	4.24 ± 0.73^b^	1.16 ± 0.00^d^
3-Methyl-6-Hepten-1-ol	N.A	N.D	N.D	N.D	1.28 ± 0.10^a^	1.31 ± 0.00^a^	N.D
3-Methyl-1,2-cyclopentanediol	N.A	N.D	N.D	3.04 ± 0.53^a^	N.D	N.D	N.D
3-Cyclohexene-1-ethanol	N.A	N.D	N.D	N.D	2.19 ± 0.11^a^	2.79 ± 0.60^a^	N.D
Alcohols (15)							
Benzene	4900	N.D	N.D	14.53 ± 0.99^b^	21.87 ± 4.06^a^	N.D	N.D
Toluene	1550	20.14 ± 4.40^a^	18.87 ± 0.73^a^	13.45 ± 0.71^b^	8.00 ± 1.56^c^	N.D	6.84 ± 0.82^c^
Ethylbenzene	2205.25	11.64 ± 4.24^a^	N.D	4.81±1.25^b^	N.D	1.06±0.02^c^	2.00±0.56^c^
*o*-Xylene	0.28	13.91 ± 7.00^b^	9.06 ± 0.99^c^	N.D	26.82 ± 10.73^a^	1.66 ± 0.11^d^	2.38 ± 0.64^d^
*p*-Xylene	0.091	N.D	N.D	N.D	3.81 ± 1.36^a^	N.D	N.D
(1-Propylnonyl)-benzene	N.A	1.66 ± 0.24^a^	N.D	N.D	N.D	N.D	N.D
Phenol	2400	1.74 ± 0.08^a^	N.D	N.D	0.90 ± 0.16^b^	1.23 ± 0.22^ab^	N.D
Aromatics (7)							
2-Ethyl-furan	2.3	134.10 ± 17.35^a^	56.42 ± 6.05^c^	110.94 ± 8.16^b^	3.78 ± 0.55^d^	4.62 ± 0.13^d^	6.73 ± 0.99^d^
2-Pentyl-furan	5.8	14.27 ± 3.13^a^	N.D	4.42 ± 0.49^b^	2.47 ± 0.77^bc^	1.97 ± 0.40^c^	0.69 ± 0.09^d^
2-*n*-Heptylfuran	N.A	N.D	N.D	N.D	N.D	1.81 ± 0.09^a^	N.D
Furans (3)							
*N,N*-dimethyl-methylamine	2.5	N.D	N.D	N.D	81.17 ± 10.90^ab^	96.03 ± 32.94^a^	77.55 ± 15.07^b^
Pyridine	2000	N.D	28.77 ± 11.49^a^	N.D	N.D	N.D	N.D
Methyl-pyrazine	N.A	1.56 ± 0.47^d^	4.46 ± 0.49^b^	N.D	3.37 ± 0.40^c^	5.81 ± 0.78^a^	N.D
1,3-Diazine	N.A	3.18 ± 0.27^a^	N.D	N.D	N.D	N.D	N.D
Pyrazine	60	N.D	5.61 ± 1.00^a^	N.D	1.31 ± 0.05^b^	2.04 ± 0.77^b^	N.D
2,6-Dimethyl-pyrazine	200	N.D	9.77 ± 1.08^a^	N.D	1.85 ± 0.31^b^	11.16 ± 3.69^a^	N.D
Trimethyl-pyrazine	1800	2.66 ± 0.00^a^	N.D	N.D	N.D	N.D	N.D
1,5-Dimethyl-1H-pyrazole	N.A	N.D	N.D	N.D	11.78 ± 0.15^a^	N.D	N.D
Pyrrole	N.A	2.43 ± 0.24^b^	7.56 ± 0.78^a^	2.66 ± 1.06^b^	1.12 ± 0.14^c^	3.19 ± 1.29^b^	N.D
N-containing compounds (9)							
Pentane	N.A	121.63 ± 36.87^c^	209.90 ± 32.82^b^	75.84 ± 15.95^cd^	518.60 ± 47.88^a^	16.72 ± 3.98^d^	11.49 ± 2.67^d^
Hexane	N.A	21.64 ± 0.31^b^	8.82 ± 2.70^d^	14.24 ± 2.50^c^	81.17 ± 10.90^a^	N.D	N.D
Heptane	730	17.92 ± 5.18^bc^	49.95 ± 2.46^a^	22.95 ± 5.26^b^	13.55 ± 1.64^c^	7.04 ± 2.35^d^	N.D
Octane	730	5.53 ± 1.71^c^	10.02 ± 1.74^b^	15.96 ± 2.89^a^	4.23 ± 0.91^c^	N.D	N.D
(*Z,Z*)-3,5-Octadiene	N.A	1.15 ± 0.39^a^	N.D	1.23 ± 0.04^a^	N.D	N.D	N.D
*D*-Limonene	10	N.D	N.D	N.D	31.32 ± 3.05^a^	N.D	N.D
1-Methyl -2-ethylcyclopentene	N.A	1.61 ± 0.61^a^	N.D	N.D	N.D	N.D	N.D
3-Ethyl-2,6,10-trimethylundecane	N.A	N.D	N.D	N.D	N.D	N.D	2.57 ± 0.38^a^
3,5,5-Trimethyl-1-hexene	N.A	11.98 ± 1.92^a^	N.D	8.74 ± 1.77^ab^	6.26 ± 1.68^b^	9.78 ± 1.55^a^	2.30 ± 0.62^c^
4-Cyanohexene	N.A	3.33 ± 0.00^ab^	2.97 ± 0.22^b^	4.09 ± 0.64^a^	N.D	1.80 ± 0.06^c^	N.D
1,7-Dimethyl-7-(4-methyl-3-pentenyl)-tricyclo[2.2.1.0(2,6)]heptane	N.A	N.D	16.07 ± 1.84^a^	N.D	N.D	7.45 ± 1.22^b^	N.D
2,6,10,14-Tetramethyl-Pentadecane	N.A	N.D	N.D	N.D	N.D	N.D	1.72 ± 0.37^a^
2,6,10-Trimethyl-dodecane	45.28	3.07 ± 0.34^c^	3.76 ± 0.29^c^	8.12 ± 1.27^a^	2.84 ± 0.39^c^	6.23 ± 0.24^b^	N.D
1-Methoxy-cyclohexene	N.A	1.60 ± 0.28^a^	N.D	N.D	N.D	N.D	N.D
Hydrocarbons (14)							
Carbon disulfide	N.A	17.11 ± 4.20^b^	37.86 ± 13.26^a^	18.69 ± 4.63^b^	4.18 ± 1.63^c^	4.20 ± 0.71^c^	N.D
Dimethyl sulfide	12.3	N.D	41.54 ± 5.69^a^	35.49 ± 9.81^a^	7.04 ± 1.36^c^	9.46 ± 1.86^c^	17.12 ± 1.19^b^
*n*-Butyl ether	N.A	N.D	N.D	N.D	1.51±0.39^a^	N.D	N.D
Disulfide, dimethyl	0.06	N.D	16.22 ± 3.03^a^	N.D	N.D	N.D	N.D
Formic acid, pentyl ester	N.A	N.D	N.D	2.54 ± 0.51^b^	21.82 ± 2.89^a^	2.72 ± 0.53^b^	N.D
Thiazole	N.A	2.04 ± 0.51^b^	13.08 ± 0.35^a^	3.30 ± 0.83^b^	2.67 ± 0.94^b^	1.72 ± 0.09^c^	1.51 ± 0.11^c^
*n*-Caproic acid vinyl ester	1	161.81 ± 10.08^a^	N.D	N.D	N.D	N.D	N.D
Propanoic acid	8.1	N.D	N.D	2.59 ± 0.16^a^	2.00 ± 0.09^ab^	1.67 ± 0.29^b^	N.D
Dimethyl sulfoxide	N.A	N.D	N.D	4.93 ± 1.27^a^	N.D	N.D	1.42 ± 0.20^b^
2(3H)-Furanone, dihydro-3-methylene-	N.A	5.64 ± 0.98^a^	N.D	N.D	1.73 ± 0.59^b^	2.47 ± 0.25^b^	N.D
Pentanoic acid	N.A	11.69 ± 1.44^b^	14.70 ± 0.08^a^	6.17 ± 0.40^c^	5.51 ± 0.07^c^	15.18 ± 1.49^a^	3.94 ± 0.58^d^
Hexanoic acid	22,000	59.54 ± 6.14^a^	30.83 ± 2.18^b^	27.68 ± 3.07^b^	14.05 ± 1.02^c^	3.27 ± 0.67^d^	2.56 ± 0.05^d^
2-Ethyl-hexanoic acid	N.A	7.22 ± 1.47^b^	12.24 ± 0.55^a^	12.98 ± 3.30^a^	3.43 ± 0.62^c^	7.43 ± 2.77^b^	2.12 ± 0.05^d^
Octanoic acid	3000	1.89 ± 0.16^a^	2.33 ± 0.21^a^	2.32 ± 0.59^a^	N.D	N.D	N.D
Nonanoic acid	3000	6.43 ± 1.30^a^	6.39 ± 0.42^a^	6.89 ± 1.37^a^	1.85 ± 0.31^bc^	2.60 ± 0.36^b^	1.25 ± 0.09^c^
Other(14)							
	Total	6128.76 ± 254.68^a^	4542.08 ± 79.50^b^	4254.88 ± 459.50^b^	1464.13 ± 166.57^d^	3091.09 ± 215.44^c^	1307.22 ± 126.17^d^

*Note:* Data are presented as the mean ± SEM (*n* = 3). Values in the same line with different letters are significantly different (*p* < 0.05).

Abbreviations: LC, lake crabs; PC, pond crabs; RC, rice-field crabs.

**Table 5:** Key volatile compounds in roe/paste from pond, lake, and rice-field environments.

Compounds	Female roe			Male paste		
	Lake	Pond	Rice-field	Lake	Pond	Rice-field
Butanal	0.71	3.98	0.40	1.19	2.88	3.63
2-Methyl-butanal	0.17	1.00	0.06	0.52	2.11	2.71
3-Methyl-butanal	0.46	4.38	0.22	1.30	10.01	6.43
Pentanal	2.12	5.84	1.17	3.40	23.25	16.47
Heptanal	2.32	3.19	1.79	1.03	1.17	0.91
Octanal	2.78	4.90	1.87	—	0.67	1.38
Nonanal	1.43	1.78	0.95	0.80	0.75	0.86
(*E*)-2-Nonenal	5.59	—	2.10	—	—	—
(*E,E*)-2,4-Octadienal	0.48	2.69	0.41	—	3.53	—
1-Penten-3-one	1.16	1.38	—	2.59	3.01	—
1-Octen-3-one	100.00	100.00	100.00	100.00	100.00	100.00
1-Octen-3-ol	1.82	3.27	1.02	4.04	2.35	2.24
*o*-Xylene	1.46	1.84	—	16.14	0.77	2.18
2-Ethyl-furan	1.71	1.39	1.12	0.28	0.26	0.75
*N,N*-dimethyl-methylamine	—	—	—	5.47	4.96	7.93
Dimethyl disulfide	—	15.36	—	—	—	—
*n*-Caproic acid vinyl ester	4.76	—	—	—	—	—

Abbreviations: LC, lake crabs; PC, pond crabs; RC, rice-field crabs.

**Table 6:** Odorants identified in roe/paste from pond, lake, and rice-field environments by GC–O analysis.

Compounds	Odor description	Female roe			Male paste		
		Lake	Pond	Rice-field	Lake	Pond	Rice-field
*N,N*-dimethyl-methylamine	Fishy	—	—	—	5.4725	4.9625	7.9268
Methanethiol	Putrefactive	—	0.3479	—	—	—	—
Propanal	Disinfectant water smell, reagent smell	—	—	0.0299	—	—	—
Butanal	Burnt meat, woody	0.7069	3.9845	—	—	—	—
3-Methyl-butanal	Reagent, cooked meat, irritating	—	4.3755	—	—	16.0207	—
2-Ethyl-furan	Cheese, sour, tofu	1.7140	1.3943	1.1187	0.2764	0.2597	0.7462
Pentanal	Reagent, sour, irritating	2.1188	5.8363	1.1727	3.4028	23.2506	16.4720
Hexanal	Herbaceous	0.0203	0.0188	0.0114	0.0051	0.0065	0.0077
Heptanal	Fruity, fresh, grassy	2.3172	—	1.7878	—	—	—
(*Z*)-4-Heptenal	Chemical, bitter	0.1079	0.0824	0.0622	0.1129	0.1253	0.0818
Octanal	Green, green leaf, tropical fruit	2.7840	4.9002	1.8669	—	—	—
1-Octen-3-one	Mushroom, cooking flavor	100.0000	100.0000	100.0000	100.0000	100.0000	100.0000
2,3-Octanedione	Grassy, exciting	—	—	0.1292	0.5427	—	0.4983
2,6-Dimethyl-Pyrazine	Milk, corn, rice	—	0.0028	—	0.0017	0.0078	—
1-Hexanol	Leafy, fresh	0.0003	0.0006	0.0002	—	—	—
(*E*)-2-Octenal	Smell of soap, laundry detergent	0.4265	0.2973	0.3113	—	0.2842	—
1-Octen-3-ol	Roast aroma, sour	1.8195	3.2706	1.0231	4.0416	2.3475	2.2436
(*E*)-2-Nonenal	Chinese medicine, cucumber	5.5911	—	2.1010	—	—	—
(*E,Z*)-2,6-Nonadienal	Cucumber	0.2184	—	0.1420	0.2292	0.1718	—
2-Butanone	Fatty	0.0029	0.0131	0.0005	0.0118	0.0065	0.0179

Abbreviations: LC, lake crabs; PC, pond crabs; RC, rice-field crabs.

## Data Availability

The data that support the findings of this study are available from the corresponding author upon reasonable request.
